# Toward a Theory of Nature Experience and Health

**DOI:** 10.1089/eco.2022.0005

**Published:** 2022-12-02

**Authors:** Linda Powers Tomasso, Jarvis T. Chen

**Affiliations:** ^1^ Department of Environmental Health, Harvard T.H. Chan School of Public Health, Boston, Massachusetts, USA.; ^2^ Population Health Sciences Program, Harvard University, Boston, Massachusetts, USA.; ^3^ Department of Social and Behavioral Sciences, Harvard T.H. Chan School of Public Health, Boston, Massachusetts, USA.

**Keywords:** Nature engagement, Nature experience, Nature alienation, Social behavioral theory, Health disparities, Urban health interventions

## Abstract

This article presents an integrated theoretical framework to study the socioenvironmental attributes of the nature experience as a basic health behavior. After first reviewing existing literature on theories behind nature exposure, we discuss social cognitive theory (SCT) to explain individual nature experience through the model's triadic dynamic of environment, cognitions, and behaviors. We then expand beyond SCT's focus on the individual to examine structural and societal spheres of influence on nature experience found in ecological systems theory and ecosocial theory. In moving from proximal to distal influences, we identify the core constructs of each theory that may reinforce or deter decisions inclining individuals toward nature engagement. In synthesizing aspects of these three theories, we propose an integrated theoretical framework of nature experience distinguished by three ideas. First, individual-level formative influences in nature pervade higher level ecologies as a learned social behavior. Second, nature experience happens within multiple systems and timepoints. Third, social relationships within historical processes shape contextual factors of the nature experience, resulting in disparities in nature access and nature responses that manifest heterogeneously. Theorizing behind nature experience can inform why this occurs. We offer suggestions for further research to build on the groundwork put forth here: for hypothesizing around present observations, for collecting data to confirm and/or refute parts of the theory, and for further hypothesis generation inspired by the theory to inform the research agenda. In conclusion, we consider the practical implications of theory underlying nature experience as a health behavior relevant to research, interventions, and policy.

## Introduction

Agrowing literature around nature contact and positive health outcomes has led to calls to expand opportunities for contact with nature, especially within cities. The importance of being in nature as a respite from urbanization has come from writers, painters, and landscape architects for >175 years. Olmsted explained mid-19th century interest in natural landscapes that developed in parallel to city expansion “as a self-preserving instinct of civilization” (Olmsted, Beveridge, & Hoffman, 1997, p. 345). More recently, scientific research has established nature as a healthful antidote to urban illnesses, for example, stress conceptualizing “nature exposure,” however, without much detail in defining “exposure.”

We can think of the predominant “nature exposure” paradigm as being influenced by how environmental exposures (e.g., pollutants in air, water, or soil) are traditionally studied. As applied to nature exposure assessment, this consists of (1) descriptions of population exposure patterns often tied to geography, (2) exploration of mechanistic pathways that describe physiological and psychological responses to nature contact, (3) experimentation with tools to refine exposure metrics and outcome magnitudes, and (4) epidemiological approaches to infer statistical relationships between exposure to nature (e.g., timing and dosage) and health outcomes.

**Fig. 1.**
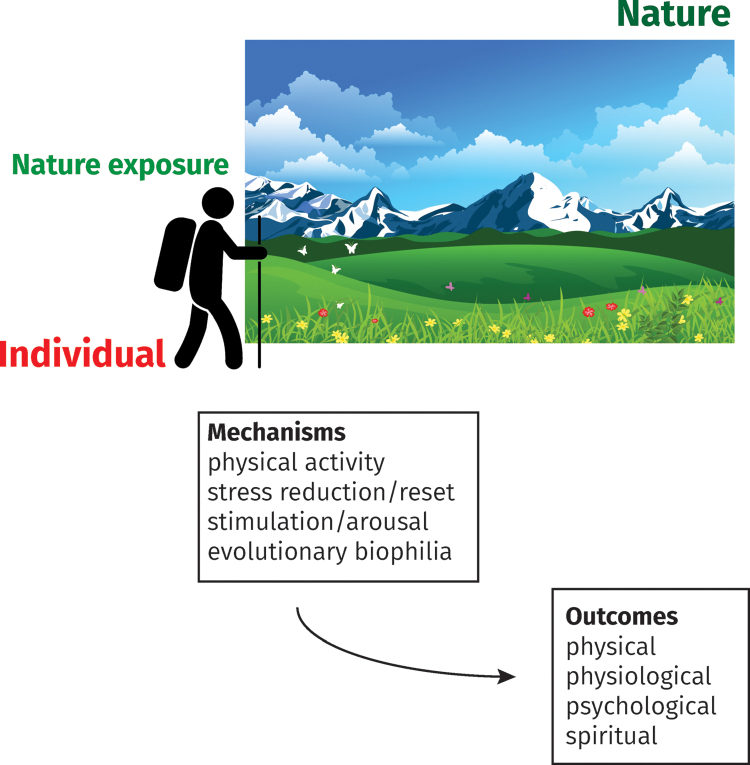
A simple individual-level exposure–outcome model for the health effects of exposure to nature. The individual who spends time in nature experiences health benefits (physical, physiological, psychological, and spiritual) from exposure to nature.

In these categories, “nature exposure” tends to be passively defined and divorced from its social context, insofar as the emphasis is on the immediate effects of greater or lesser nature contact (Fig. 1). Without a fully contextualized theory of the nature experience, however, we argue that the discipline places limits on how scientific knowledge can inform efforts to affect the complex behaviors that underlie nature exposure or, indeed, to appreciate the deeper phenomenological richness of what constitutes nature experience.

A deeper theoretical treatment of “nature” must first begin with a richer set of definitions, which the extreme subjectivity of nature and nature use demands. To allow for maximum inclusiveness, we define nature along multiple axes—indoors versus outdoors, limited versus panoramic, sentient versus nonsentient, abstract versus familiar, intentional versus incidental—overlain with scales of size, wildness, and approachability. However, we also distinguish between the qualities of nature exposure and the potential benefits that an exposed individual may derive from it.

For example, the apartment dweller tending houseplants may appear objectively less nature immersed than the weekend landscape hiker and yet may be as equally nature engaged despite different activity outlets. A strictly quantification approach to nature exposure might, therefore, miss a relevant subjective appraisal of the nature experience, as well as overlook the possibility that subjective factors themselves are conditioned by precursors to current nature engagement. Acknowledging that individual-level factors may moderate physiological and psychological reactions to nature importantly relaxes a uniform response model for all population subgroups (Richardson & Mitchell, 2010). Exploring reasons for heterogeneous responses to nature is a potential implication of nature-seeking theories.

We also distinguish passive nature contact from active nature engagement, with implications for intentionality and reciprocity in the relationship with nature. Intentionality characterizes the nature experience for many nature users (Reese, Hadeed, Craig, Beyer, & Gosling, 2019) but should not be considered a requisite of benefit. The literature concedes that intentional nature engagement differs from incidental nature contact (Keniger, Gaston, Irvine, & Fuller, 2013; Martin et al., 2020; Richardson, Hamlin, Butler, Thomas, & Hunt, 2021) but gives health-promoting evidence for both exposure types (Dadvand & Nieuwenhuijsen, 2019; Shanahan et al., 2016; White, Pahl, Wheeler, Depledge, & Fleming, 2017; Wyles, White, Hattam, Pahl, & King, 2017).

Therefore, accepting nature and nature seeking as multifaceted, subjective concepts leaves space for the development of a richer theory of nature experience, where “experience” encompasses passive contact, intentional engagement, nature affinity, and nature-seeking.

A theoretical framing of the nature experience must also consider social and behavioral determinants that enable access and exposure (Birn, 2009; Commission on the Social Determinants of Health, 2008; Krieger, 2008). At issue is who interacts with nature and why, and how social structures facilitate or inhibit those interactions. An awareness of the inequitable barriers to nature exposure alerts us to the salience of nature disengagement and alienation as distinct dynamics. Although studies have documented many health inequities related to spatially patterned nature access (Casey et al., 2017), theory is also needed to understand the psychological determinants of nature disengagement and alienation that may compound effects above and beyond merely the absence of nature contact.

Nature deprivation strongly associates with negative physical and emotional health (Chawla, 2015; Kahn & Kellert, 2002; Thompson, Aspinall, & Montarzino, 2008) just as nature exposure supports positive health gains (Dean et al., 2018; Pretty, 2004; Pretty, Peacock, Sellens, & Griffin, 2005; Thomsen, Powell, & Monz, 2018). Lack of nature contact is considered detrimental to emotional and psychological development, appearing to limit full cognitive and emotional development in children (Thompson et al., 2019; Ziljema et al., 2017). If true, researchers and those who look to research for its practical implications need to consider both ends of the nature contact scale, nature deprivation and engagement. An encompassing theory should identify nature alienation as the underside of nature engagement with its probable determinants.

Nature engagement is moreover a set of learned behaviors that take place in culturally specific contexts, such as use of nature for social recreation, medicinal/curative ends, and aesthetic enjoyment, so that inclinations to seek out nature may themselves be socially conditioned (Bell, Phoenix, Lovell, & Wheeler, 2014). Socially conditioned contexts for behavior hold importance for understanding how and why people interact with nature. Although researchers on nature exposure and health implicitly tend to approach a desire for nature contact as universal, there is literature that suggests that nature affinity in fact varies across individuals and is malleable even (Ito, Leung, & Huang, 2020; Schultz, 2002; Schultz & Tabanico, 2007).

Putting aside the question of whether a uniform affinity for nature exists, it seems likely that the combination of life experiences as well as interactions with social structures that constrain opportunities for nature contact and nature appreciation affects the manifestation of nature affinity in individuals.

To develop a rich theory of nature experience, with attention to the issues of nature definition, social and behavioral context, structural obstacles, and learned social behaviors requires drawing on various disciplines with explanatory value: for example, psychology, sociology, ecology, and urban and policy planning. Nature experience exemplifies Antonovsky's ecological understanding of health as buttressed by general resistance resources, for example, (Antonovsky, 1996). The goal of this article, therefore, is to look critically at theoretical models that describe the multiple facets of nature experience.

The behavioral underpinnings of nature engagement align well with the constructs of social cognitive theory (SCT). Bandura considered his SCT well suited for planning suitable public health interventions (Bandura, 1998), an assessment sustained by previous applications of this theory to public health (Clark & Zimmerman, 1990; Rosenstock, Strecher, & Becker, 1988; Sharma, Wagner, & Wilkerson, 2005; Steinhardt & Dishman, 1989).

We expand upon SCT's focus on the individual to examine the ecological and historical context for nature seeking through Bronfenbrenner's ecological systems theory (EST) and Krieger's ecosocial theory (ET). We believe that a rich theory of nature experience benefits from the wider conceptual analysis these three theories bring. By integrating concepts from these theories, we seek to describe the scope, structure, and opportunities presented by an emergent integrated theory of nature experience and open its further elaboration to researchers in the nature–health field.

## Dominant Paradigm in Nature–Health Research

The nature exposure paradigm prevailing over five decades of research has formalized the health benefits of nature contact. Evidence from the investigative community has associated nature exposure, and urban greenspace particularly, with positive health endpoints, including reduced all-cause mortality (Gascon et al., 2016; James, Hart, Banay, & Laden, 2016; Rojas-Rueda, Nieuwenhuijsen, Gascon, Perez-Leon, & Mudu, 2019; Vienneau et al., 2017), reduced mental illness (Barton & Pretty, 2010; Bratman et al., 2019; Walsh, 2011), and fewer inflammatory disease markers (Andersen, Corazon, & Stigsdotter, 2021; Kuo, 2015; Li, 2010; Rook, 2013).

Allergic, inflammatory, and autoimmune diseases, for example, asthma and depression, occur at higher rates in urbanized societies (Flies et al., 2019; Phillips, 1993), and physiological stressors such as urban heat gain, limited spaces for exercise, and poor air quality concentrate in densely built environments (Chakraborty, Collins, & Grineski, 2016; Kabisch, 2019; Kabisch, Korn, Stadler, & Bonn, 2017). This has led to calls for expanding opportunities for exposure to nature, especially for urban populations.

Although mutedly acknowledging that interaction underlies exposure, the existing nature exposure paradigm only inferentially questions who interacts with nature and why. What remains short is an actionable theoretical framework through which scientific knowledge can combine with a phenomenological understanding of nature exposures to advance health for all.

Some well-known theoretical frameworks already explain observed human responses to nature exposure: attention restoration theory (Kaplan, 1995), stress reduction theory (Kaplan & Kaplan, 1989; Ohly et al., 2016; Stevenson, Schilhab, & Bentsen, 2018), and biophilia (Kellert & Wilson, 1993; Wilson, 1984; Woodworth, 2021). These mainly anthropocentric theories situate humans apart from rather than within an ecological framework (Schweitzer, Glab, & Brymer, 2018). Place attachment theory, in contrast, locates the individual within an environment, natural or otherwise, to explore the forging of emotional bonding and identity (Giuliani, 2003).

Environmental injustice examines inclusivity in nature through cultural–historical facts of land ownership and access (Byrne, 2012; Draus, Haase, Napieralski, Roddy, & Qureshi, 2019; Taylor, 1999; Tozer, Hörschelmann, Anguelovski, Bulkeley, & Lazova, 2020), a forceful theoretical framework pertaining to certain but not all groups. Lachowycz and Jones (2013) present a social–ecological framework hypothesizing causal pathways between greenspace access and health by focusing on potential moderating and mediating factors, given the difficulty of quantifying socioenvironmental factors. Schweitzer et al. (2018) apply phenomenological methods to build their theory of psychological identification with nature as significant for human well-being.

However, none of these theories apart from environmental injustice explores the sociobehavioral process behind the nature experience. Perhaps the closest theoretical approach to nature seeking is the multitheory model (MTM) used to account for determinants of behavioral change for initiating intentional outdoor nature contact routines on college campuses (Sharma et al., 2020). However, the abundance of greenspace characterizing college campuses limits the generalizability of MTM to explain behavioral engagement with nature outside this specific context.

## Impetus for Theory Development

The impetus for this article was our observations in two qualitative studies we conducted (Tomasso, Cedeno, Chen, & Spengler, 2021; Tomasso, Cedeño Laurent, Chen, Catalano, & Spengler, 2021) that nature experience as described in the prevailing scientific literature is undertheorized in comparison with the vivid narratives we collected from participants in our focus groups. In relating their lived experience, nature users we studied described complex relationships with nature that had roots in formative experiences in childhood, often mediated by significant figures.

Emergent themes that arose in our phenomenological investigation of personal experiences (Teherani, Martimianakis, Stenfors-Hayes, Wadhwa, & Varpio, 2015) included intentionality of nature seeking, individual agency to pursue nature at odds with reliance on organizational support, structural deficiencies in transport and utility supporting nature use, leading us to recognize the need for a bigger framework of active nature engagement.

Inductive thematic analysis of synthesized focus group comments made clear that exposure to nature was shaped by layered influences proximal and distal, familial and societal, generational, locational, and normative as well as heterogeneous expectations and expectancies from past experiences in nature. This prior study grounds our abductive approach to building an integrated behavioral theory of nature engagement in this article (Timmermans & Tavory, 2012).

## Materials and Methods

The study was conducted according to the guidelines of the Declaration of Helsinki and approved by the Institutional Review Board of Harvard T.H. Chan School of Public Health (protocol code 19-1419 on 28 August 2019).

## Theoretical Model Presentations

### Social cognitive theory

We introduce SCT as the primary theoretical grounding among the prevailing psychological theories of behavioral health to describe nature engagement and nature alienation as learned social behaviors. The many dynamics embedded in the SCT model constructively illustrate how individuals acquire personal capabilities for engaging in targeted behaviors such as autonomous nature pursuit. The model's signature dynamic, “triadic reciprocal determinism,” examines how personal cognitions and environmental influences mutually interact with and balance the target behavior (Bandura, 1986; Wood & Bandura, 1989).

SCT draws from social behavioral science for its two theoretical frameworks to explain individual behavior formation: antecedents to factors motivating behaviors and antecedents to modifiers of behavioral responses (Bandura, 1986; Luszczynska & Schwarzer, 2015). SCT emphasizes self-efficacy as the primary predictor of intention critical to behavioral change (Bandura, 1969). In fact, much of the impetus behind the SCT's effectiveness credits self-efficacy made possible by cognitions, acquired skills, and self-regulation to perform behaviors successfully (Zimmerman, 1990).

For this reason, SCT is essentially agentic in nature, meaning individuals retain an ability to develop and exercise a capacity for self-efficacy toward changing behavior, provided that appropriate environmental reinforcements and collective normative and social support are in place (Bandura, Ross, & Ross, 1963; Perry, Baranowski, & Parcel, 1990).

The theory's key motivational components are expectations, expectancies, and cognition of personal skills and qualities necessary for goal attainment. These three social–cognitive variables together have proven useful in predicting behavior (Bandura, 1986; Barone, Maddux, & Snyder, 1997; Fiske & Taylor, 1991) and are relevant to nature engagement. Expectations can sum up the anticipatory physical, social, and self-evaluative outcomes of nature-oriented behavior, for example, social acceptance in an outdoor group. Individuals who experience the outcomes expected from nature will find their motivations for continuing that behavior reinforced by actualized expectations and cognitively supported by self-feedback.

By contrast, expectancies for nature contact denote a measure of value the individual places on anticipated outcomes, for example, apprised well-being or satisfaction from goal attainment, and are central to spurring nature engagement under the SCT framework. Expectancies shape future experiences (Maddux, 1999). According to Bandura, all social–cognitive theories include a behavior–outcome expectancy that connects a specific behavior to a specific outcome in a specific situation, making expectancies around behavior one of the most important components of cognitive construal strategies (Maddux, 1999). Contextualizing current nature-oriented behaviors as the outcome of successful past experiences rechannels future expectations and expectancies for time in nature (Table 1).

**Table 1.** Application of Social Cognitive Theory Concepts in Relation to Nature-Seeking Behaviors^*^

CONCEPT	COMPONENTS	DEFINITION	IMPLICATION FOR NATURE SEEKING
Environment (reinforcements)	Vicarious modeling Experiences Physical environment Natural environment Family, friends Other social influences Social norms Organization/institutional support	Factors physically external to the person that reinforce one's ability to access nature successfully and derive health benefits from experience Ex. Successful foray outdoors with friends leads to mutual satisfaction	Natural environment: proximity of wild nature, urban greenspace. Physical: neighborhood safety and accessibility, infrastructure, efficient transit Organizational support: church and summer camps, scouts, ecology clubs
Situation	Combines objective facts (physical risk, weather, other people) with subjective reading (hostility, welcome, phobias)	Person's perception of the environment: safe, welcoming, hostile, manageable, transparent? Ex. perceived microaggressions in public nature spaces	Imparting knowledge of objective factors, e.g., safety vs. risk + building self-efficacy through practice and praise Conceptions of nature highly influenced by vicarious modeling
Behavioral capability	Actionable skills Reactions Practice Protective conditions in interim	Knowledge and skill to perform a targeted behavior, here going into nature Ex. Navigating a blazed trail	Take what was learned and observed by shifting onus of behavior from support to oneself, i.e., lead or go alone
Expectations	Reasons for nature seeking modified by prior experiences	Anticipatory outcomes of a behavior Ex. Stress release from everyday grind	Better physical/emotional health, restorativeness, happiness, solitude, awe and wonder, stress removal; longevity, weight loss
Expectancies	Preferences/tastes, excitement, high failure bar, safety, belief in the health benefits of outdoor recreation/nature exposure, stress reduction, relaxation all motivate behavior	The (quantifiable) values that one places on a given outcome; emotional (dis)incentives Ex. Fear of disorientation in woodlands (negative); experiencing awe (positive)	Change from domestic environment, recreation, escape from computer/office environment; enabling conditions to think (solitude, quiet), beauty
Self-control	Self-monitoring Self-regulation	Personal regulation of goal-directed behavior or performance Ex. Limit thrill seeking to what is safe	Developing judgment of one's own ability, knowledge when to retreat, perseverance in challenge
Observational learning (vicarious modeling)	Someone to model—relative, teacher, camp counselor Group dynamics Instructional learning of both event and outcome	Behavioral acquisition that occurs by watching the actions and outcomes of others' behavior Ex. Know how to read sky conditions for impending weather change	Presence of a trusted adult who introduces nature in a positive way, models behavior and outcome expectation, and transfers skills to identify and control risks outdoors
Reinforcements	Positive incentives: feeling healthy, achieving goals, better sleep, strength, social network Negative incentives: animals, bad weather, scary persons, boredom, excessive fatigue	Responses to a person's behavior that increase/decrease the likelihood of repeating the behavior Ex. Receive affirmative information and encouragement toward goal	Hedonic well-being (short-term, ex. prize earned) vs eudemonic well-being (self-reinforcing, longer-term, ex. cardiovascular health)
Self-efficacy (primary predictor of intention)	(Highly important b/c affects how much effort invested in tasks and level of performance attained.) Feedback, social support, self-rewards	Person's confidence in performing a particular behavior and in overcoming barriers to that goal; seek specificity about change Ex. Visualizing information in context	Mental preparation, conditioning, perseverance, see goal to end. Recognition of risks, preparation, and management of risks to avoid real danger. Learned by vicarious modeling
Emotional coping response	Opportunities to practice skills in emotionally arousing situations	Experiences will modify strength of response to social/cognitive behavior Ex. Learning not to over-react to failure	Satisfaction with task completion vs. discouragement that experience was disappointing, boring, hostile
Barriers	Time, money, transport. Energy, effort; competing interests, screen time; knowhow, sense of security, weather. Inconvenience; lack of time.	Barriers that a person perceives toward enacting/adopting the behavior Ex. Developing ability to foresee barriers and devise alternatives	If barriers perceived as too high/not worth the effort to overcome, person will not seek outdoor activities, thus increasing nature alienation

^*^ Inspired by Perry, Bar, Parcel (1990).

SCT evolved from Bandura's early conceptualization of social learning (Bandura, 1969; Bandura & Walters, 1963; Zimmerman & Schunk, 2003). Social learning theories recognize that expectations are learned by prior experience, particularly performance attainment, vicarious observation of others' experience, and reactions to those experiences, as well as participatory learning after observation (Bandura & Walters, 1963; Bandura et al., 1963; Manz & Sims, 1981). Social learning in nature is fundamental; nature enthusiasts and professional conservationists alike describe having been positively socialized to nature by an influential parent or older sibling, teacher, or organizational leader (Chawla, 2007; Chawla & Derr, 2012).

Social learning and social processes account for the variability in nature-seeking propensities, motivations to initiate time outdoors, and likely responses in nature. Social persuasion and one's own emotional/physical responses to nature engagement reinforce social learning processes. Vicarious observation cognitively rewards the individual with feedback, thus inclining the individual to repeat the learned behavior when confronted with familiar situations in nature (Bandura, 2001). Observation and modeling of nature-oriented behaviors permit novices to become acculturated to the affordances and risks in natured environments that are difficult to learn outside direct experience.

Autonomous nature seeking presumes an underlying knowledge and comfort outdoors, behavioral capabilities, and obtainable rewards from engaging with nature. These multiple cognitions are given license to develop within contexts of unfamiliar natural environments with sufficient environmental and behavioral support. Enabling environments, both in wild nature and urban greenspace, favor the acquisition of the skills, confidence, and assessment competencies to be able to access and use nature safely. In fact, nature engagement rests heavily on the environmental component implicit in SCT's triadic balance.

Environmental reinforcements are essential for cultivating adult nature-seeking behavior and may include modeling and mentorship, prior experiences, supportive social norms, and enabling physical and natural environments. However, weaker reinforcing factors (places, people, and organizations) that nurture nature-inclined cognitions and behaviors inhibit the development of autonomous nature seeking, straining SCT's triadic equilibrium.

SCT's focus on the individual perhaps overemphasizes self-efficacy as the most important proximal determinant of nature engagement. The theory saves room for collective agency as a form of environmental reinforcement, but that burden still falls to a degree on individuals' circumstantial ability to organize. Reinforcing contexts open doors to collective action for counteracting less supportive conditions, though nature users may not be consciously aware of the constellation of SCT factors influencing their learned behaviors while nevertheless being affected by them.

Moreover, for many, time in nature infers the opposite of finding peace in nature—fear of injury, lack of resources, experiences such as shaming (self- or external) over skill inability or anxiety, or structural obstacles such as transportation to reach natured environments–and may reduce self-efficacy. Limitations of individual decision making around nature engagement, therefore, need also be examined from a wider context of history, norms, and systemic levels. SCT also describes an individual's environment somewhat vaguely, even though nature seeking is actualized in the individual's sociocultural environment. We thus turn to EST to provide historical–normative context to behavioral influences on nature experience.

### Ecological systems theory

We begin to articulate a new paradigm of nature-seeking behavior by grouping Bronfenbrenner's EST with undertheorized by parts of SCT. EST has enjoyed wide application to public health for its concern with the “ecology of human development” (Bronfenbrenner, 1979, 2005). Ecology relates organisms or groups of organisms to their environment (Krieger, 2011, p. 209), each in separate dynamic flux. Bronfenbrenner's father, a medical doctor with a doctorate in zoology, oriented the future developmental theorist to ecological systems witnessed in the distinct ongoing development of individuals vis-à-vis their member group.

“Wherever we walked, he would alert my unobservant eyes to the workings of nature by pointing to the functional interdependence between living organisms and their surroundings” (Bronfenbrenner, 1945, p. 608). Indeed, this childhood realization that hierarchically nested structures operate as systems in themselves and in relation to each other influenced Bronfenbrenner's conceptualization of EST. Bronfenbrenner's later renamed *bioecological paradigm* (Belsky, 1995; Bronfenbrenner, 2005) accounted for this biological nexus between developing individuals and evolving environments.

Many operational features of nature experience fall within the “ecological context of influential relationships” specified by EST (Bronfenbrenner, 1985). A defining property of the paradigm states that human development takes place through processes of progressively more complex reciprocal interaction between an evolving individual and the persons, objects, and symbols of the immediate environment. These interactions are referred to as “proximal processes.”

Proximal processes substantively and theoretically bear upon the person–environment behavioral interaction (Bronfenbrenner, 1995; Bronfenbrenner & Evans, 2000; Merçon-Vargas, Lima, Rosa, & Tudge, 2020) in ways that direct the cultivation of behaviors toward or away from nature-based recreation, socialization (Bronfenbrenner et al., 1996; Espelage, 2014), and restorative well-being. For example, a person uninitiated to nature may be favorably or unfavorably susceptible to the belief systems of parents, teachers, mentors, spouses, and close friends regarding nature and environmentalism. Table 2 summarizes EST's main influences on the ecology of nature experience.

**Table 2.** Components of Ecological Systems Theory as They Relate to Nature-Seeking Behavior

CONCEPT		COMPONENTS	DEFINITION (+ EXAMPLE)	IMPLICATION FOR NATURE SEEKING
Individual		Self Perception of self	Active agent of influence on the environment Ex. Ability and comfort felt in nature	Degree of agency in and toward nature affected by broader ecosystem
Individual's ecosystem	Microsystem	Home, immediate family, school/work environment, peers, neighborhood, etc.	Defined by three “engines of development”: interaction with persons, objects, and symbols Ex. Family has weekly picnics outdoors	Most influential and proximate level of ecosystem; developmental transference vis-vis nature
	Mesosystem	Inter-relates home, schools, neighborhood, work, etc.	Sphere that connects elements of exosystem to microsystem Ex. Parent's workload precludes time outdoors yet finds no organizational support	Interdependence of microsystems provides fluid context of opportunities and (non-) support for nature seeking
	Exosystem	Mass media, community services, school or workplace, culture, membership organizations	Other formal systems that do not explicitly contain nature-seeking individuals but have indirect influence Ex. Quality of urban parks and transit to access them dis/favors engagement	Potential nature seeker relies on group, messaging, and infrastructure networks to activate behavior
	Macrosystem	Shared cultural values, beliefs, customs, and laws, socio-economic status, ethnicities, location	Already-established culture and society the nature seeker is in Ex. Nature seeker feels welcome vs. dismissed outdoors	Reception by wider society in nature spaces will dictate if nature seeker feels safe or atrisk outdoors
	Chronosystem	Environmental events Life transitions Historical events	Environmental changes occurring over lifetime that influence development Ex. Weakened place attachment with time	Transitions may enable or disenable individual from pursuit of nature seeking
Ecological transition		Developing person, ecological environment	When person's position in ecological environment is altered in response to changes in role, setting, or in characteristics of developing person Ex. Successful navigation in wild	Confronting phobias with success (snakes, darkness, disorientation)
Reciprocal activity		Participation of the developing person, Significant individual who facilitates learning and development	Developing person participates in progressively more complex patterns of reciprocal activity with person of trust and enduring attachment Ex. More novice partner plans and leads outdoor route	Shifts balance of power in favor of developing person
Transforming experiment		Social status, social structures Individual, group, development process	Systematic alteration and/or restructuring of existing ecological systems to challenge belief systems, forms of social organization, and lifestyles of given (sub)culture Ex. More inclusive leadership of conservation organizations	Opposes extant systems or creates new structures
Proximal processes		Evolving human, plus Persons, objects, symbols	Interaction between an actively evolving human and the persons, objects, and symbols of immediate environment Ex. Females alone in woodlands	Effect reduced if environment in which processes occur is unstable
Timing of biological and social transitions		Culturally defined age Role expectations Opportunities Passage of time	Relates to the culturally defined age, role expectations, and opportunities across one's life course Ex. Taking *your* kids into nature	Influences the course and outcome of development of those closest to you

EST overlaps with the social learning modeling posed by SCT; Bandura calls this vicarious observation or reciprocal activity, Bronfenbrenner vicarious modeling. In both models, the development outcomes at one age become the personal characteristics that influence development outcomes at a later age (Bronfenbrenner, 1995), a response the literature on nature engagement supports (Thompson et al., 2008). EST uniquely adds an enveloping chronosphere that captures the effect of environmental events associated with nature and their timing occurring over the individual's lifetime. Ecological and social transitions express changes over time.

Under the life-course perspective, the timing of ecological and social transitions accounts for the culturally defined age, role expectations, and opportunities occurring throughout one's life and, therefore, extensively influences the course and outcome of behavioral development. We are currently witnessing how social media, video gaming, and 24/7 screen-based interaction are transitioning the “exosystem” for most individuals that competes directly with nature seeking (Kelly & Coughlan, 2019). Such technological developments distinctly dampen nature inclinations, particularly among children. In specifying a chronosphere external to all nested layers, EST can remain nimble to modern developments that impact efforts to reach nature, preserving the theory's interpretive relevance since its 1970s introduction.

While attending to the individual through self-perception and levels of nested influence, EST undertheorizes formal and informal structures that explain distributed patterns of health and well-being. Using this theory to make broad assumptions about individuals is ill advised, as nested hierarchies are but one form of ecological structure. Not only do non-nested hierarchies exist, but also hierarchical forms themselves are not “fixed,” depending instead on concurrent biological processes (Krieger, 2011, p. 205). Still, EST undertheorizes the structural aspects of nature engagement in relation to inequity and cultural sets, while perhaps overemphasizing the developmental contribution of the environment. We thus shift from EST to integrate ET into our nature experience paradigm.

### Ecosocial theory

Stepping further back, Krieger's ET (Krieger, 1994) examines the epidemiology of health phenomena in relation to their societal, ecological, and historical contexts. This model emphasizes that societal processes drive the social patterning of health and disease distributions and pays great attention to nested levels of societal organizations as impacting conditions for health outcomes like those derived from nature experience. ET not only accommodates but *requires* consideration of the inherent links between proximal and distal levels of influence on individual health (Krieger, 2011, p. 205; Vygotsky, 1978) such that societal and biological features are dynamically interlaced at every level of the ecosocial design (Krieger, 2011, p. 210).

An ecosocial approach to nature seeking embeds health benefits derived from greenspace use within strata of influence promoting or neglecting population health. The model foresees how supportive public infrastructure such as park maintenance and transit can assist adoption of positive behaviors such as physical activity, and how geolocated data on pediatric health, housing density, and food assistance should spatially inform nature Rx programmatic interventions. Acknowledging these often distal but interactive levels of influence allows nature experience to be construed as a societally structured opportunity. Concluding otherwise can lead to oversimplifying nature exposure as a matter of time and distance.

ET preserves both the individual's connection to society and the individual uniqueness within socially defined categories when evaluating health prospects and outcomes. The model treats pathways of health embodiment literally such that benefits cumulatively accrue through exposures like nature just as risks from harmful exposures like pollutants accumulate (Table 3). These exposure disparities persist over generations, and race and socioeconomic status compound exposure outcomes.

**Table 3.** Core Constructs of Ecosocial Theory. Opportunities Affecting Individual Health Reflect Historical Policy, Norms, Community Contexts, and Institutional Structures Governing Them as Pertain to Nature Experience^*^

CONCEPT	COMPONENTS	DEFINITION (+ EXAMPLE)	IMPLICATION FOR NATURE SEEKING
Embodiment	Societal conditions Dynamic ecological state Group relations Cultural practices and beliefs	Bodily engagement (soma and psyche combined), individually and collectively, with the biophysical world and each other Ex. Nature affinity, connectivity	Do present societal and ecological dynamics enable or prevent the embodiment of health and well-being?
Pathways of embodiment	Social and economic (de)privations Social affordances and/or discrimination Health care Ecosystem health	Diverse, concurrent, and interacting pathways through which positive and negative exposures occur Ex. Locally accessible urban park quality signaling neighborhood investment	If socially structured links between nature exposure and health outcomes vary over time and place, then here-to-fore standards may not suffice
Cumulative interplay of exposure, susceptibility, and resistance	Embodied exposures Conditioned responses Gene–environment interactions	Manifest patterns of embodied exposures accumulate, respond to, and are timed to historical and geographical contingencies Ex. Therapeutic equivalent of forest immersion for persons of color	If habituation to nature contact fortifies “instorative” resiliency, can nature seeking be made appropriately habitual?
Accountability and agency	Levels of power Individual's capacity to act (agency) responsibility	The capacity for action at each level to address social disparities in health along with research to explain health inequities Ex. Realigning governance and mission of conservation societies to reflect all users	What programmatic and planning changes need to occur to equitize nature-seeking opportunities? At what level is the locus of agency for nature access?
Lifecourse	Historical context Generations	Historically specific and spatially patterned rates and trends of health outcomes; appropriately long timeframes for analysis and interpretation Ex. Longitudinal health studies in denatured urban cores	What are best means to redress current nature access that resulted from historical processes?
Processes	Production Exchange Consumption Reproduction	Current and changing societal arrangements of power, property, and production and reproduction of both social and biological features at every level	Is nature truly a public good—or is nature seeking explained by processes of production, supply, and demand?

^*^ Inspired by Krieger, 2011.

Nature alienation also acts as a pathway of negative health embodiment through social and economic deprivation, historical legacy, and ecosystem degradation, which all tend to accumulate with time. Although not deterministic, ET acknowledges fewer opportunities exist to produce favorable nature-related health outcomes in certain built environments.

Distal influences have immediate relevance to nature experience. From a structural perspective, ecosocial theorists might ask whether we need more history—within societies, within polities, and within families—to understand nature experiences. Historical aspects modulate over time, though longer exposures tend to entrench existing attitudes; how will long-term land dependency translate to a recreational image of the outdoors?

From a biological basis, we can deepen inquiry of gene expression within nature-based settings; how might formative experiences beneficially condition nature exposure outcomes in adulthood? From a societal context, messaging around nature engagement is inconsistently framed and interpreted; does social signaling instill a sense of agency or provoke ecoanxiety around rights, access, and safety in natured spaces? We must ask whether nature alienation reflects only greenspace access or conditioned avoidance.

### Theoretical model integration

**Fig. 2.**
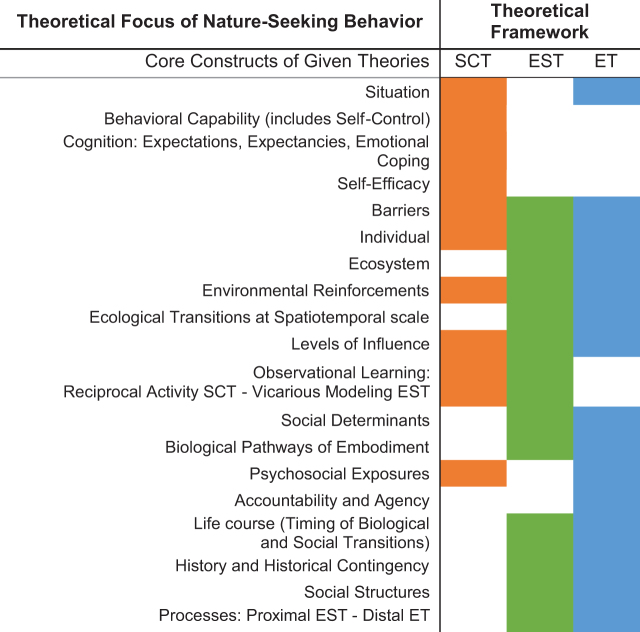
A comparison of the key constructs for each sociobehavioral theory describing nature-seeking, with each color representing a theory: SCT, social cognitive theory; EST, ecological systems theory; ET, ecosocial theory.

Gathering these three sociobehavioral theories, we present an integrated theoretical model informed by our qualitative data to support theory generation around nature experience (Fig. 2). The juxtaposition of constructs from each theory accentuates how key theoretical elements, respectively, cluster in a left-to-right diagonal around individual, ecological, and social/historical/temporal levels of influence. Theory integration helps explain nature exposure less as a function of dose–response than as a behavioral continuum ranging from circumstantial alienation to active engagement. Three ideas distinguish our integrated nature experience theory from standard hypothesizing around nature exposure.

First, individual-level formative influences in nature, fundamental to soldering life-long nature engagement, enter higher level ecologies as a learned social behavior. Second, nature experience happens within multiple systems and timepoints but within limited sites and geographies, for example, urban neighborhoods. Third, social relationships within historical processes shape contextual factors of the nature experience, whereas structural inequalities among disadvantaged groups perhaps stymie them. These processes result in disparities in nature access and responses to nature, once accessed, which can manifest heterogeneously.

## Discussion

The nature–health discipline warrants a theoretical modeling of sociobehavioral factors behind nature exposure adequate to 21st century society. Our study considered the ability of three theories to explain the nature experience as molded by individual-centered factors operating within a nested ecosystem at intertwined structural layers. We present this theoretical framing of nature engagement and disengagement as learned behaviors as a critique of the existing nature exposure paradigm.

Much is known about health outcomes of nature contact, though theoretical models outside psychology have largely overlooked the structuring of socioenvironmental underpinnings of nature experience, even as operational support for nature engagement both exists and has been critically examined in the literature as being effective. Successful examples are Nature R_x_ programs, Girls Scouts, and Outward Bound. Studies of organizational platforms enabling nature engagement, however, often fall outside the exposure assessment discipline and tend to overlook agentic development central to these programs.

**Fig. 3.**
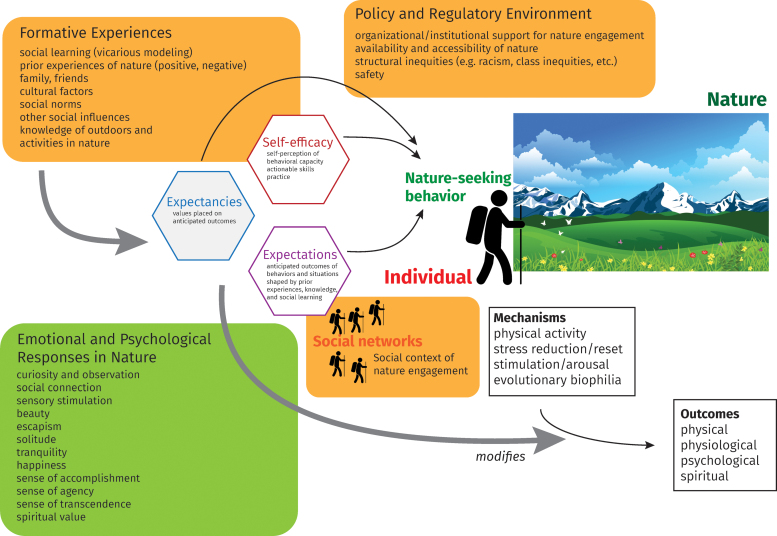
A more complex ecosocial and sociobehavioral model that contextualizes the antecedents of nature experience and their impacts on the nature–health relationship. The individual's nature experience is shaped by formative experiences and their impact on expectations, expectancies, and self-efficacy. The nature experience takes place within a social context defined at multiple levels by one's immediate social networks, the community, and the policy and regulatory environments. Expectations, expectancies, and self-efficacy modify mechanisms and outcomes through emotional and psychological responses in nature.

Although individual choices to engage with nature affect health, choices never occur in a vacuum. Nature experience explained through SCT alone reveals the limits of individual choice and agency. The EST hierarchy situates behavioral nature engagement within a multilevel ecological context unfolding over time. The yet more distal ET examines how opportunities to act out one's nature-seeking inclinations are determined by relevant social structures (Bronfenbrenner & Ceci, 1994). The theories together furnish a broader context of theoretical analysis to consider the deeper societal and historical structures and policies of nature experience for the individual (Fig. 3).

A theory-driven approach to assess nature contact for the purpose of catalyzing nature experience might pause to consider multiple levels of influence posed by our integrated model, which appropriately addresses both nature alienation and nature engagement. The theory also accommodates the steady retraction of two traditional on-ramps to nature: formative places and formative persons. These introductory experiences generally occur in childhood in proximity to significant outdoor places, providing opportunities for creative play in solitude or under the guidance of trusted individuals. Urbanization and population densification have squeezed out the first foundational influence formative places in nature.

Children today rarely grow up near formative nature-based environments where they can play independently vis-a-vis previous generations. They also encounter less biodiverse, more homogeneous urban nature, and fewer affordances for imaginative interaction outdoors. The reach of formative places itself has narrowed to exclude not just wild outcrops near one's home but also formerly visited farms, gardens, or large backyards of grandparents where children learned nature skills, food origins, and lessons of growth and resilience. Family dispersion and working parents with less time for nature activities explain the second retreating influence of nature experiences: formative adults.

We found that positive exposure to nature has precedence in formative persons who comfortably introduce and safely acculturate individuals to nature-based environments. Together, the two formative influences of places and persons constitute strong behavioral reinforcements. Removing one or both in childhood erects barriers to the intuitive pull of natured environments later for adults, leading to indifference, fear, or aversion toward a potentially health-promoting exposure. In addition, feelings of repulsion due to historical memory and cultural experiences transpired in nature become more difficult to reverse in adulthood.

### Implications

The implications of our integrated theory begin with directions for further investigating the determinants of nature experience. Deconstructing the behavioral underpinnings of nature engagement and, conversely, nature alienation is paramount and informative for translating theory into practice. Social contexts shown to facilitate or inhibit nature engagement may point to the design of interventions that account for distal and proximal influences on nature-seeking behavior well beyond exposure. Proposed directions for research, interventions, and policy build on our integrated theory of healthful nature experience.

### Research implications

Environmental reinforcements to experience nature at the individual level assume not only accessible natured spaces but also opportunities for modeled behavior. Mentorship involves modeling demonstrated skills and risk management outdoors, expressions of care toward living organisms in nature, and a recognized value of time in nature over alternative pursuits. Pathways to nature as learned in prior generations may no longer hold if elements supporting nature-seeking behaviors such as mentorship are missing.

There are limits to what personal factors can accomplish if the suite of environmental reinforcements is comparably weak. Intervention studies might, therefore, analyze the weights that various environmental reinforcements exert on actualizing nature inclinations to mime influences in broader ecological contexts. Participatory action research also can learn what resources and priorities exist in nature-poor neighborhoods to better support nature interaction.

Longitudinal studies tracking nature exposure from childhood into adulthood are essential to identify key age windows where individuals are most impressionable to the physiological and emotional imprint of nature interaction. An appropriate framework for such an ambitious assessment might be a nested ecological study design with time-variant dynamics related to age and emotional maturation that shape person-level desire and capability to engage in nature. As children develop and mature, levels of influence expand within their ecosystems. Learning at which levels leverage rests for introducing children and adolescents comfortably to nature and instill behaviors favorable to long-term nature engagement can be useful for ongoing health interventions such as nature prescriptions and outdoor peer leadership.

Such interventions that encourage positive nature experiences can fit well into a study design informed by EST. Organizations that foster long-term nature engagement such as Scouts might provide research contexts and study populations to track changing agentic and motivational dimensions of nature experience, for example, expectancies and self-efficacy, not only markers quantitatively associated with nature exposure such as decrease in Body Mass Index, academic improvement, or hours spent outdoors.

### Intervention implications

What is the appropriate role of the researcher in proposing interventions? Community health requires assessment, and SCT prescribes explicit factors for designing interventions that motivate and sustain nature engagement as a target health behavior. Behavioral interventions should aim to replicate core constructs instrumental to nature engagement identified by SCT, notably strategies that enhance self-regulation, self-efficacy, outcome expectations, and expectancies for nature exposure (Umstattd & Hallam, 2007). As the long-standing entryways to autonomous time in nature narrow, programmatic interventions must assume the role of eliciting and sustaining nature-oriented behaviors.

Free-standing initiatives to increase nature contact such as supplying free park passes—to national parks particularly—may fall short without supportive contexts. As societal changes render vicarious modeling relationships and backyard environments less common, public health interests must also advocate for renewed spaces to explore nature, for example, forest preschools, hybrid schoolyards incorporating food gardens and pond ecosystems, and new mentorship, for example, teachers in outdoor classrooms, to take their place. Who designs these interventions is as important as the interventions themselves, with venues, programs, and terms of engagement to be cocreated and endorsed by user groups, designers, and implementors within communities.

### Policy implications

Since urban nature shows strong geographic patterning, the social determinants of healthful nature experience become spatialized around greenspace access. Policies to redress the unequal distribution of urban greenspace typically approach nature access through urban development or planning. Yet development approaches too often result in green gentrification, hence defeating their intended purpose. Integrated sociobehavioral theory instead suggests community support of nature engagement, as health outcomes reflect social gradients rooted in community disparities.

The social epidemiological literature points to neighborhood-level effects on health disparities. Since social exposures reflect neighborhood-level distribution, policies might approach more equitable nature experience through social networks, social risk taking, and available local institutions and resources, not urban development alone.

Building an inclusive outdoor culture in which nature experience is structurally unrestricted taps into ET. Increasing nature engagement at multiple levels of intervention will require structurally expanded modes of access so that nature seeking can plant ecological roots in every sense. Distal but pervasive influences of institutional support, re-examination of cultural norms, that is, in/exclusion, structural realignment toward nature accessibility, and cooperative stakeholder action must be leveraged toward nature equity. Ecosocial or sociobehavioral models that rely heavily on social contexts and self-regulation may seem unessential at earlier stages of nature exposure assessment but become critical for proposing corrective legal guidelines at later implementation phases.

As example, conservation organizations such as the Appalachian Trail Conservancy and the Sierra Club have embraced equity and inclusion as governance principles, serving as examples of institutional mission change. Ecosocial examples abound in public health research (Hsieh, Apostolopoulos, Hatzudis, & Sönmez, 2016; Nardone et al., 2020; Walters et al., 2011; Zierler & Krieger, 1997) and effectively explain how institutional and regulatory positions can redress socioeconomic disparities of health. Treating nature as a mediator of health rather than an urban amenity can begin to translate the idea of nature engagement into effective policy.

## Conclusion

This article examines reasons why nature exposure has escaped theoretical scrutiny as a beneficial health behavior and offers an integrated behavioral model to fill this gap. The new paradigm we propose considers social determinants of nature experience at multiple levels and encourages a rethinking of nature contact in behavioral terms rather than uniquely exposure terms. Narrowing urban health disparities through nature engagement is a multidimensional prospect. Our integrated theory of nature experience broadens the theoretical context of nature exposure, retreating from individual to more distal and often generational factors of influence. Layering interventions as complementary leverage points for personal, programmatic, and policy interventions can help individuals discover and access their fuller health potential in nature.
